# A comparison of different human papillomavirus tests in PreservCyt versus SurePath in a referral population—PREDICTORS 4

**DOI:** 10.1016/j.jcv.2016.06.015

**Published:** 2016-09

**Authors:** Jack Cuzick, Amar S. Ahmad, Janet Austin, Louise Cadman, Linda Ho, George Terry, Michelle Kleeman, Lesley Ashdown-Barr, Deirdre Lyons, Mark Stoler, Anne Szarewski

**Affiliations:** a Centre for Cancer Prevention, Wolfson Institute of Preventive Medicine, Queen Mary University of London, Charterhouse Square, London EC1M 6BQ, UK; b Department of Colposcopy, St Mary’s Hospital, Imperial College Healthcare NHS Trust, London W2 1NY, UK; c Department of Pathology, University of Virginia Health System, Charlottesville, VA 22908, USA

**Keywords:** Human papillomavirus testing, Cervical screening, PreservCyt, ThinPrep, SurePath

## Abstract

**Background:**

Two transport media, PreservCyt and SurePath, are widely used for cervical cytology screening. There are concerns that they may perform differently for HPV testing.

**Objectives:**

A comparison of the performance of six different HPV tests in SurePath and PreservCyt in a referral population using two samples from each woman. The primary goal was to compare the performance of each test in the two media. Comparisons between assays and viral load comparisons between media were secondary aims.

**Study design:**

Two cervical samples were collected in random order at the same visit in women with abnormal cytology. One sample was placed in 20 ml of PreservCyt and the other in 10 ml of SurePath. Aliquots were taken for 4 DNA based tests: *digene* HC2 High-Risk HPV DNA Test, Abbott Realti*m*e, BD Onclarity and Genera PapType, an RNA based test—: Hologic Aptima and a protein test: OncoHealth.

**Results:**

630 sample pairs were included in the analyses. For all tests except the protein test sensitivities were in excess of 90% for CIN2+ and 95% for CIN3+ for both media and with no significant differences except for a lower sensitivity for CIN2+ of Aptima in SurePath (93% vs 98%, P = 0.005). Specificity for <CIN2 was significantly better in Surepath for HC2, RealTi*m*e and Aptima, and generally lower relative signal strengths were seen with SurePath except for Onclarity, especially when it was the second sample.

**Conclusions:**

We found similar sensitivity for CIN3+ in PreservCyt and SurePath for 5 nucleic acid tests in the two media in a referral population, but signal strength and positivity rates were lower in SurePath except for the Onclarity test. These results need to be replicated in a screening population.

## Highlights

• First comparison of HPV tests in PreservCyt and SurePath, 2 samples from each woman.
• Nucleic acid HPV tests showed similar performance in PreservCyt and SurePath.
• Manufacturers’ recommended pre-treatment protocols must be observed.

## 1 Background

Two liquid-based cytology (LBC) systems are commonly used: ThinPrep using PreservCyt transport medium (Hologic Inc., Marlborough, MA) and SurePath (Becton Dickinson, Sparks, MD) using SurePath Preservative Fluid. Slide preparation procedures from these media are different [1], [2]. Cells are normally collected using a Cervex-Brush (Rovers Medical Devices, Oss, Netherlands) but in PreservCyt cells are rinsed into the medium, dispersed by vortexing, and transferred to a microscope slide after vacuum filtration. In SurePath, the detached head of the brush is placed in the medium. After initial centrifugation cells are resuspended, put through a density gradient centrifugation with sampling of the pellet to make the slide. The performance of both systems for cytology is comparable [1], [3].

An advantage of LBC is that additional tests, notably HPV, can be run from a single sample, although only PreservCyt is approved by the FDA for this. Unlike PreservCyt, SurePath contains formaldehyde to preserve cell morphology and cross-linkage between protein and nucleic acid can occur which can make DNA undetectable and reduce DNA yield. This is partially reversible using proteinase K (PK) digestion and/or heat treatment prior to nucleic acid purification [4], [5], [6]. It is currently unclear whether such treatment can provide sufficient native HPV DNA/RNA from individual cervical samples for different HPV assays.

The majority of early studies of HPV testing in a medium also suitable for cytology have been conducted using Qiagen’s *digene* HC2 High-Risk HPV DNA Test (HC2) in PreservCyt. In a study of 972 SurePath and 1033 PreservCyt screening samples in different women Zhao et al. found no significant difference in sensitivity and specificity for the detection of CIN2+ by HC2 [7]. In a Danish study of 5064 screening samples the positivity rate was found to correlate moderately well (kappa ≥0.60) between four assays (HC2 (Qiagen), Cobas (Roche), CLART (Genomica) and Aptima (Hologic)) using SurePath and multiple testing on one sample from each woman [8]. In another study of 367 women with abnormal cytology this group reported similar sensitivities for these four assays [9]. The UK Sentinel Sites study of 10,051 women referred with borderline or mild dyskaryosis showed a higher overall HPV positivity rate in PreservCyt than SurePath (68.7% vs 61.7%, p < 0.0001). However this may be confounded by site as all but one site used only one medium and the site using both media found no significant difference in positivity rates [10]. To our knowledge, there has not been a comparison of the performance of different HPV assays using PreservCyt and SurePath samples collected from the same woman.

## 2 Objective

The objective of this study was a comparison of the performance of different HPV testing assays in SurePath and PreservCyt in a routine clinical setting. We used a colposcopy referral population and compared six HPV assays using two samples from each woman—one collected in PreservCyt and the other in SurePath. Our primary goal was to compare the performance of each test in the two media. Comparisons between assays were secondary aims.

## 3 Study design

The study was conducted in the Colposcopy Unit of St. Mary’s Hospital, London among women who had been referred with an abnormal screening result within three months and never treated for CIN. All women provided written informed consent.

Two cervical samples were collected with Cervex-Brushes immediately prior to colposcopic examination in accordance with the European guidelines for quality assurance with cervical cancer screening [11]. To minimise bias, the order of use of transport medium was randomly assigned (1:1). One brush was agitated in a vial containing 20 ml of PreservCyt. The other brush head was removed and deposited in a vial containing 10 ml of SurePath. All samples were stored at 4 °C and transferred within two weeks of collection to the laboratory at the Wolfson Institute of Preventive Medicine, where HPV testing was performed.

Within one day of receipt in the laboratory, samples were warmed to room temperature, agitated for 60 s and aliquotted into a fixed order set of tubes, appropriate for six assays. This was pseudo-randomised to vary the aliquot assigned to each assay by using one of four dispensing patterns (left to right, right to left, centre to right then centre to left, centre to left then centre to right). Samples were only identifiable to laboratory staff by participant number. All pathology was reviewed by M.S. who was blinded to results and participant information.

## 4 Laboratory methods

Sample storage before testing, aliquot volumes and positivity cut-off values were all in accordance with the manufacturers’ instructions (Table 1). No tests were done on post-gradient pellets.

Manufacturers use ‘Invalid’ or ‘Indeterminate’ to denote failed results including when a whole plate or run fails. We refer to all as ‘Failed’ results in this paper.

### 4.1 Assays

#### 4.1.1 DNA based

• *digene* HC2 High-Risk HPV DNA Test: the QIAsymphony automated platform was used for nucleic acid extraction with the DSP AXpH DNA Kit (Qiagen, Hilden, Germany). This consensus DNA test detects a panel of 13 high-risk HPV types (16,18,31,33,35,39,45,51,52,56,58,59,68). PreservCyt and SurePath samples were processed using different protocols: PC_AXpH_hc2_V1_DSP protocol and a modified SP2000_V1_DSP protocol including PK digestion and extended heated lysis time (provided by Qiagen for research purposes only) respectively [12]. 4 ml PreservCyt or 0.5 ml of SurePath diluted in 2 ml of deionised water were used. The resulting eluates (60 μl) were dispensed into a 96 well microplate for manual testing. Signal strength was measured in Relative Light Units (RLU) compared to a reference of approximately 5000 HPV copies.
• The Abbott RealTi*m*e High-Risk HPV assay used the m2000 processing System (Abbott Molecular, Abbott Park, Illinois) for the detection of 14 high-risk HPV types, utilising Abbott reaction vessels as sample input tubes. Types 16 and 18 are individually reported. The remaining 12 high-risk types are reported together as a pool (31,33,35,39,45,51,52,56,58,59,66,68).
• The Becton Dickinson Onclarity HPV Assay using the BD Viper LT System is a real-time PCR based DNA test which detects 14 high-risk HPV types. Types 16,18,31,45,51,52 are detected individually. The remaining eight high-risk types are reported in three groups: (33,58), (35,39,68) and (56,59,66). A 0.5 ml aliquot of thoroughly vortexed SurePath or PreservCyt was added to 1.7 ml of a proprietary HPV diluent. A heat step was employed to ensure that exfoliated cells were lysed and the sample homogenized prior to extraction of sample DNA [4], [5].
• The Genera PapType Test is a semi-automated, bead-based multiplex full genotyping DNA assay for 14 high-risk HPV types (16,18,31,33,35,39,45,51,52,56,58,59,66,68) and two low-risk HPV types (6,11). The Sirocco platform (Genera Biosystems, Scoresby, Australia) was used. Prior nucleic acid extraction was done using the Abbott m2000sp instrument [13]. Only high-risk types were considered positive in this study. The assay measure is derived from flow cytometry and reported as S (signal). Type specific cut-offs were used (Table 1).

#### 4.1.2 RNA based

• The Hologic Aptima HPV assay is based on target capture, transcription-mediated amplification and hybridization protection for the detection of E6/E7 mRNA expression of 14 high-risk HPV types (16,18,31,33,35,39,45,51,52,56,58,59,66,68). A consensus result for positivity to other high-risk types was provided. The Direct Tube Sampling platform was used. Typing for 16 and 18/45, available as a reflex test, was not done here. The SurePath sample was treated with PK at 65 °C for 2 h before being assayed manually. The cut-off was specified to be 0.5 of the ratio of the intensity to the reference standard.

#### 4.1.3 Protein based assay

• OncoHealth (OncoHealth, San Jose, California) protein test is a direct E6/E7 HPV Whole-Cell ELISA carried out in microtitre wells and is based on detection by non-type specific HPV E6 and E7 monoclonal antibodies [14]. Relative Optical Density (ROD) was used compared to a reference value of 0.35.

For all HPV tests except HC2 both samples were processed using an identical assay workflow. (To distinguish this from workflow associated with sample preparation). Test details using Preservcyt for HC2, Onclarity, RealTi*m*e, PapType and Aptima have been described previously [13], [15], [16], [17].

## 5 Statistical analysis

The primary analyses consisted of paired comparison of the two samples from each woman. For some assays confounding was observed related to the order in which the sample was taken. Subsequently additional non-paired analyses by the Wilcoxon Ranksum test and a robust L1 based linear model with allowance for test order were also conducted [18]. A measure of viral load (log(1 + relative intensity units (RIU)) or minus Ct values) was used to perform correlation and regression analyses with adjustment for sample order for paired samples within each test. Here RIU refers to the signal strength of the sample compared to a standard (Table 1). Non-amplified samples for Onclarity and RealTi*m*e were given a Ct value of 40 and signal strength 0 for Aptima. SAS (version 9.2) and R (version 3.2.2) were used. All statistical tests were two-sided and a p-value of 0.05 were accepted as statistically significant.

## 6 Results

**Fig. 1:**
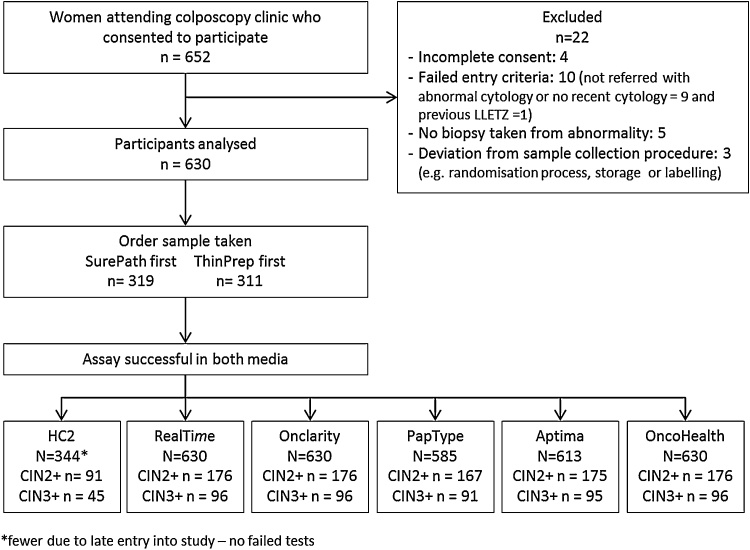
CONSORT diagram of patient enrolment and number with HPV testing by different tests.

The analysis was based on 630 sample pairs obtained from 652 participating women. Reasons for drop out are shown in Fig. 1. The median age was 30.0 years (IQR = [27.0, 34.8]). HC2 was introduced during the study, and only the last 344 sample pairs were tested. There were no failed results for HC2, Onclarity, OncoHealth or RealTi*m*e. For PapType one sample pair was not tested with either medium and 46 tests (44 sample pairs) failed (16 from PreservCyt, 30 from SurePath). For Aptima there were 22 failed tests (17 sample pairs; 10 in PreservCyt, 12 in SurePath).

Entry cytology was borderline dyskaryosis 193(30.6%), mild dyskarosis 380(60.4%), moderate dyskaryosis 37(5.9%) and severe dyskaryosis or glandular abnormality 20(3.2%). A total of 176(28.0%) histology results were CIN2 or worse, including 94(15.0%) cases of CIN3 or CGIN and 2(0.3%) cases of invasive cancer. (Supplementary Table S1).

**Fig. 2:**
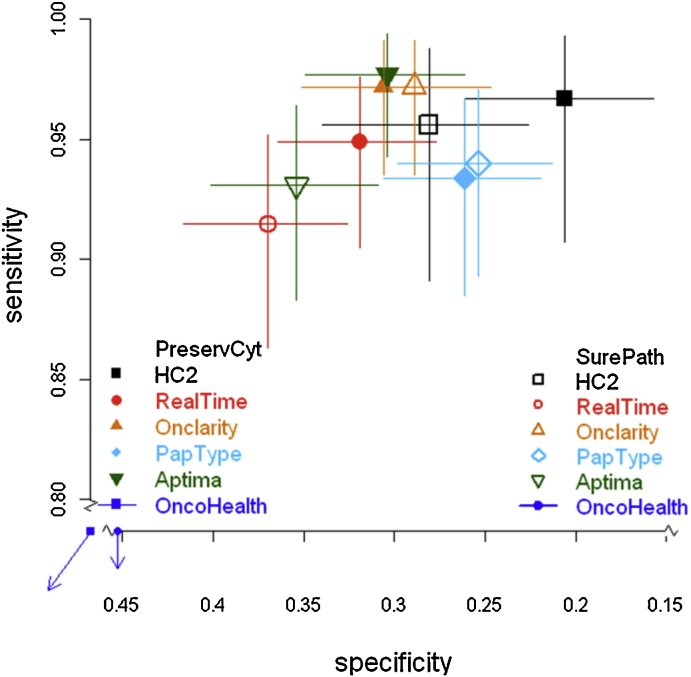
Sensitivity and specificity for CIN2+ (with 95% CIs) by HPV test and transport medium. Solid shapes show PreservCyt and open shapes are for SurePath.

Overall positivity, sensitivity for CIN3+ and CIN2+ and specificity for <CIN2 for the different tests and transport media are shown in Table 2. Sensitivity and specificity for CIN2+ are further illustrated in Fig. 2 and CIN3+ in Supplementary Fig. 1. All tests showed high sensitivities for both samples in excess of 90% for CIN2+ and 95% for CIN3+, except OncoHealth which had low sensitivity in both media. A matched-pairs analysis indicated no significant difference between media for sensitivity for either CIN2+ or CIN3+ for any test, except for Aptima which was slightly less sensitive in SurePath (98% vs 93%, P = 0.005). However, there were differences in specificity with significantly higher specificities for HC2, RealTi*m*e, Aptima and OncoHealth in SurePath. Although showing some predictive ability above chance in PreservCyt, the OncoHealth test was substantially and significantly less sensitive than all other tests (≤60% for both media for both CIN2+ and CIN3+), but was more specific than the other assays. There was no significant difference however with the OncoHealth assay between media.

Signal strength (viral load estimate) differed by transport medium and test order (Table 3). Little difference was seen between the two media when used as a first test, except for substantially higher values for RealTi*m*e in SurePath (P < 2 × 10^−5^) and HC2 in PreservCyt (P = 0.009). For HC2 this probably reflects a larger sample volume for PreservCyt. Significantly higher values were seen for PreservCyt (vs SurePath) when both were used as a second sample especially for HC2—again with the exception of Onclarity. Type specific results for HPV 16 and 18 for RealTi*m*e, Onclarity and PapType gave a similar pattern (Table 3).

PreservCyt values were not statistically significantly different between the first versus second samples in all cases except for OncoHealth where they were substantially lower in the second sample (Table 3). For SurePath, significant differences between the first and second sample were seen for all tests, but the second sample gave higher levels for RealTi*m*e and Onclarity and lower levels for the other tests. For HC2 the RLU values were much lower in the second sample for SurePath, possibly due to the smaller sample volume assayed.

The correlation between the signal strength measurements for the two media for each test is shown in Table 4. While correlations for the tests in the two media were quite good, except for the OncoHealth test, the slopes were significantly less than unity for all tests except HC2 where it was 0.966 (p = 0.23), indicating that the values are generally higher for PreservCyt. Minimal correlation between media could be seen for the OncoHealth test. A fuller presentation of the differences between the two media is shown as scatterplots for each test in Supplementary Figs. S2–S7, in which the order of the test is also depicted.

## 7 Discussion

Our results indicate that similar sensitivities and specificities can be achieved with either PreservCyt or SurePath for 5 of the 6 HPV tests, provided that the manufacturer’s recommended pre-treatments are observed. Some loss of sensitivity for CIN2 was seen for RealTi*m*e and Aptima in SurePath, but this was minimal for CIN3+. The largest differences were seen for specificity which was generally better for SurePath, especially for HC2, RealTi*m*e and Aptima. This is likely to also be true for primary screening but direct verification in this setting is needed. Poor performance was seen for the OncoHealth protein test in both media. This protein-based test however is known to be less stable in alcohol and a second generation test has been developed since this study was carried out.

The failure rate for PapType was relatively high (3.6%, 45/1260). No specific reason could be identified, but this was a prototype test with the complexity of full typing, so improvements are likely in the future. The failure rate was 1.3% for Aptima, but there were no failures for any other tests.

The differences between tests were greater for the second than the first sample, illustrating the differences in a true diagnostic situation where only a first sample would be used. This highlights the need for an adequate sample and may be a factor in the discordant results between assays as found by Rebolj et al. [8]. The SurePath vial contained 10 mls and PreservCyt 20 mls of transport medium. Thus the concentration of cells in SurePath is greater than in PreservCyt. The only test where the amount of DNA in the tested sample would be expected to be the same in both media would be the Aptima test where the aliquot volume was 1 ml of PreservCyt and 0.5 ml of SurePath. All others tests except HC2 used an equal aliquot volume (0.5 ml) and would lead to less DNA in the PreservCyt sample. For HC2 4 mls were assayed from PreservCyt versus 0.5 ml from SurePath. However this had no measurable impact on the results.

Although not of direct clinical relevance, comparison of the quantitative measures of signal strength as a surrogate measure of viral load provides additional insight into the comparative performance of the different tests in the two transport media. We recognise that there are several confounding factors to this measure including cell number and specific methods of measuring signal strength. In general lower signal strength values were obtained for SurePath. The largest differences were seen for HC2 which could partly be attributed to a smaller sample volume for SurePath.

Most HPV assays have been more fully optimized for PreservCyt, which has been in use for longer. An exception is the Onclarity assay, developed by the manufacturer of SurePath. The Onclarity assay uses a heat step in sample pre-processing for both sample types and little difference between media was seen. At the time of this study no HPV test manufacturer had an approved protocol for their assay in the SurePath medium and it is possible that this will impact on performance.

In summary this prospective study is the first comprehensive comparison of a range of HPV tests in the two most commonly used LBC transport media where two samples are taken from each woman. No major differences in performance were seen when the manufacturer’s protocols were used. These tests have all performed well in this referral population and although all appear suitable for screening. They need to be validated using the Arbyn criteria in a screening population [11].

## Conflicts of interest

Funding: funded from Cancer Research UK Programme grant C569/A16891, and supplemented by financial contributions and assay kits from Qiagen, BD, Abbott, Genera, Hologic and OncoHealth.

## Competing interests

JC has received honoraria for lectures from Abbott and Qiagen and served on advisory boards for Hologic and BD.

All authors have attended meetings with manufacturers of HPV assays but none was compensated for their work on this project.

All manufacturers had the right to comment on a draft version of this manuscript, but had no involvement in the final content or decision to publish.

## Ethical approval

Received in August 2011 from NHS Health Research Ethics Service Committee London–Hampstead [Reference 11/LO/1147].

## Figures and Tables

**Table 1:** HPV assays performed, positivity cut-off and aliquot volume.

Test	Positivity Cut-off^a^^,^^b^	Aliquot volume (ml)	
		PreservCyt	SurePath
HC2	≥1 RLU	4.0	0.5
RealTi*m*e	≤32 Ct	0.5	0.5
Onclarity	≤34.2 Ct	0.5	0.5
PapType	HPV58 ≥ 0.0004 HPV68 ≥ 0.0003 All others ≥ 0.0002	0.5	0.5
Aptima	≥0.5 RIU	1.0	0.5
OncoHealth	≥0.35 OD	1.0	1.0

a For all tests except RealTi*m*e and Onclarity, units are ratio of signal strength to reference standard.

b RLU, relative light units; Ct, cycle threshold; RIU, relative intensity units; OD, optical density.

**Table 2:** Overall positivity, sensitivity for CIN3+ and CIN2+, specificity for < CIN2 and agreement for different tests and transport media.

	Overall positivity (%)	Sensitivity		Specificity
		CIN3+ (N = 96)^c^	CIN2+ (N = 176)^c^	<CIN2 (N = 454)^c^
HC2 (N = 344)				
PreservCyt	289 (84)	0.98	0.97	0.21
SurePath	269 (78)	0.98	0.96	0.28
Agreement (%)	89.5	95.6	94.5	87.7
Discordant^a^	28 vs 8	1 vs 1	3 vs 2	25 vs 6
P-value^b^	0.001	1	1	0.001

RealTi*m*e (N = 630)				
PreservCyt	476 (76)	0.99	0.95	0.32
SurePath	447 (71)	0.97	0.91	0.37
Agreement (%)	93.8	95.8	94.3	93.6
Discordant^a^	34 vs 5	3 vs 1	8 vs 2	26 vs 3
P-value^b^	2.4 × 10^−6^	0.62	0.11	1.5 × 10^−5^

Onclarity (N = 630)				
PreservCyt	486 (77)	1.00	0.97	0.31
SurePath	494 (78)	1.00	0.97	0.29
Agreement (%)	97.1	100	100	96
Discordant^a^	5 vs 13	0 vs 0	0 vs 0	5 vs 13
P-value^b^	0.10	1	1	0.10

PapType (N = 585)				
PreservCyt	465 (79)	0.96	0.93	0.26
SurePath	469 (80)	0.96	0.94	0.25
Agreement (%)	93.5	93.4	95.8	92.6
Discordant^a^	17 vs 21	3 vs 3	3 vs 4	14 vs 17
P-value^b^	0.63	1	1	0.72

Aptima (N = 613)				
PreservCyt	476 (78)	100	0.98	0.30
SurePath	446 (73)	0.99	0.93	0.35
Agreement (%)	90.2	100	95.4	88.1
Discordant^a^	45 vs 15	0 vs 0	8 vs 0	37 vs 15
P-value^b^	1.3 × 10^−4^	1	0.01	0.003

OncoHealth (N = 630)				
PreservCyt	356 (57)	0.58	0.60	0.45
SurePath	301 (48)	0.55	0.52	0.54
Agreement (%)	55.4	46.9	49.4	57.7
Discordant^a^	168 vs 113	27 vs 24	51 vs 38	117 vs 75
P-value^b^	0.001	0.78	0.203	0.003

a PreservCyt+/SurePath- vs SurePath+/PreservCyt-.

b McNemar’s test.

c Number refers to the whole population of N = 630. See Fig. 1 for reduced numbers for HC2, PapType and Aptima.

**Table 3:** (A) Median signal strength (viral load) by test, transport medium and order of the test for samples from women positive for at least one medium using the specified test. Units are the ratio to a reference sample except for RealTi*m*e and Onclarity which are CT values. Type specific results for HPV 16 and 18 (where available) are shown in the lower part of the table. (B) 2-sided P-values for comparisons between different media and order using unpaired comparisons by the Wilcoxon RankSum test for samples positive for at least one medium.

(A) Median signal strength (RIU or CT)				
	Medium and order of sampling			
HPV Test	PreservCyt 1st	PreservCyt 2nd	SurePath 1st	SurePath 2nd
HC2	235.03	292.80	90.54	53.08
RealTi*m*e	21.30	22.03	23.64	25.85
Onclarity	24.16	24.37	23.16	24. 32
PapType	30.53	27.43	25.79	19.54
Aptima	10.67	10.81	10.55	9.80
OncoHealth	1.04	2.10	1.00	1.78
RealTi*m*e 16	20.17	22.05	24.22	24.43
RealTi*m*e 18	23.09	21.90	23.12	26.91
Onclarity 16	25. 16	25.66	24.11	24. 75
Onclarity 18	27.42	25.79	25.46	26.63
PapType 16	28.43	32.28	27.83	19.88
PapType 18	13.97	5.76	13.67	11.25

(B) Significance levels (2-sided)				
HPV Test	PreservCyt 1st vs PreservCyt 2nd	PreservCyt 1st vs SurePath 1st	SurePath 1st vs SurePath 2nd	PreservCyt 2nd vs SurePath 2nd
HC2	0.998	0.009	0.011	1.04e-07
RealTi*m*e	0.092	1.83 × 10^−5^	2.2 × 10^−8^	8.6 × 10^−15^
Onclarity	0.104	0.033	0.011	0.182
PapType	0.167	0.034	0.004	6.0 × 10^−4^
Aptima	0.313	0.094	0.012	4.7 × 10^−7^
OncoHealth	1.44 × 10^−25^	0.155	3.8 × 10^−22^	1.2 × 10^−4^
RealTi*m*e 16	0.029	1.9 × 10^−4^	0.038	2.0 × 10^−5^
RealTi*m*e 18	0.859	0.414	0.024	0.004
Onclarity 16	0.353	0.123	0.208	0.101
Onclarity 18	0.781	0.174	0.314	1.000
PapType 16	0.460	0.810	0.078	0.015
PapType 18	0.857	0.754	0.512	0.967

**Table 4:** Spearman's ρ Correlation coefficient and slope when SurePath values are regressed on PreservCyt values using L1 (robust) regression where values are either the log (1 + RIU value) or (minus) Ct value and sample order is accounted for. (See methods section). One tailed p-values compare observed slope to unity (no difference in viral load between media).

HPV Test	N^a^	Spearman’s ρ (95% CI)	Slope (95%CI); P-value (vs unity)
HC2	297	0.814 (0.771, 0.849)	0.966 (0.875, 1.057); p = 0.231
RealTi*m*e	481	0.724 (0.678, 0.764)	0.823 (0.724, 0.923); p = 2.5 × 10^−4^
Onclarity	499	0.884 (0.8.64, 0.902)	0.841 (0.778, 0.903); p = 3.0 × 10^−7^
PapType	486	0.756 (0.715, 0.792)	0.871 (0.780, 0.963); p = 0.003
Aptima	491	0.683 (0.633, 0.727)	0.676 (0.514, 0.838); p = 4.5 × 10^−5^
OncoHealth	469	−0.133 (−0.221, −0.043)	0.242 (0.121, 0.362); p < 2.010^−16^
RealTi*m*e 16	159	0.574 (0.460, 0.670)	0.653 (0.400, 0.906); p = 0.004
RealTi*m*e 18	55	0.660 (0.478, 0.787)	0.649 (0.242, 1.056); p = 0.046
Onclarity 16	161	0.838 (0.786, 0.879)	0.827 (0.677, 0.977); p = 0.012
Onclarity 18	57	0.890 (0.820, 0.934)	0.833 (0.561, 1.105); p = 0.114
PapType 16	166	0.771 (0.701, 0.826)	0.942 (0.839, 1.046); p = 0.137
PapType 18	88	0.748 (0.638, 0.828)	0.914 (0.735, 1.094); p = 0.175

a Positive at least for one test.
