# Supplementary material for: A comparison of different human papillomavirus tests in PreservCyt versus SurePath in a referral population—PREDICTORS 4
Source: J Clin Virol. 2016 Sep;82:145–51. doi: 10.1016/j.jcv.2016.06.015 (PMC4994427; doi:10.1016/j.jcv.2016.06.015)

**Supplementary Table S1. Referral smear and worst reviewed histology**

| Referral cytology (N) | Worst histology (N) | | | | | | | |
| --- | --- | --- | --- | --- | --- | --- | --- | --- |
|  | Normal colposcopy no biopsy | Inade-quate | Normal | CIN1/ HPV only | CIN2 | CIN3 or CGIN | Invasive carcinoma | Total (% N) |
| Borderline dyskaryosis  No HPV triage | 19 | 1 | 69 | 18 | 10 | 13 | 0 | 130 (20.6) |
| Borderline dyskaryosis  (HPV +ve) | 5 | 1 | 27 | 17 | 10 | 3 | 0 | 63 (10.0) |
| Mild dyskaryosis  No HPV triage | 52 | 5 | 134 | 67 | 48 | 32 | 0 | 338 (53.7) |
| Mild dyskaryosis  (HPV +ve) | 4 | 0 | 12 | 16 | 4 | 6 | 0 | 42  (6.7) |
| Moderate dyskaryosis | 1 | 1 | 1 | 3 | 6 | 25 | 0 | 37  (5.9) |
| Severe dyskaryosis/  glandular | 0 | 0 | 0 | 1 | 2 | 15 | 2 | 20  (3.2) |
| Total (%N) | 81  (12.8) | 8  (1.3) | 243  (38.6) | 122  (19.4) | 80  (12.7) | 94  (14.9) | 2  (0.3) | 630  (100.0) |

**Supplementary Figure S1. Sensitivity and Specificity for CIN3+ by HPV test and transport medium.**

**Solid shapes show PreservCyt and open shapes are SurePath**


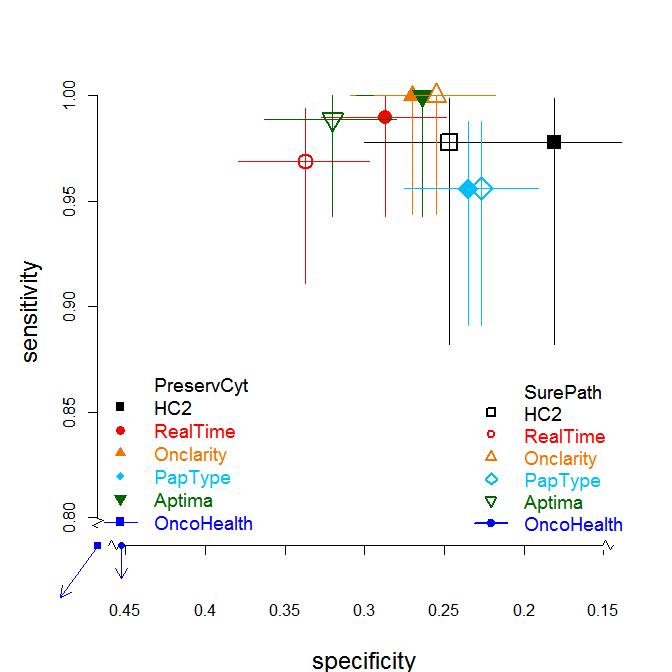


**Supplementary Figure S2. Scatterplot of *digene* HPV Test RLU values for all tested samples. The solid line is the regression line for SurePath regressed on PreservCyt adjusted for sample order. The dashed line is the 45 degree line. Samples with PreservCyt first are open and those with SurePath first are solid. Shaded area indicates values below the positivity cut-off.**


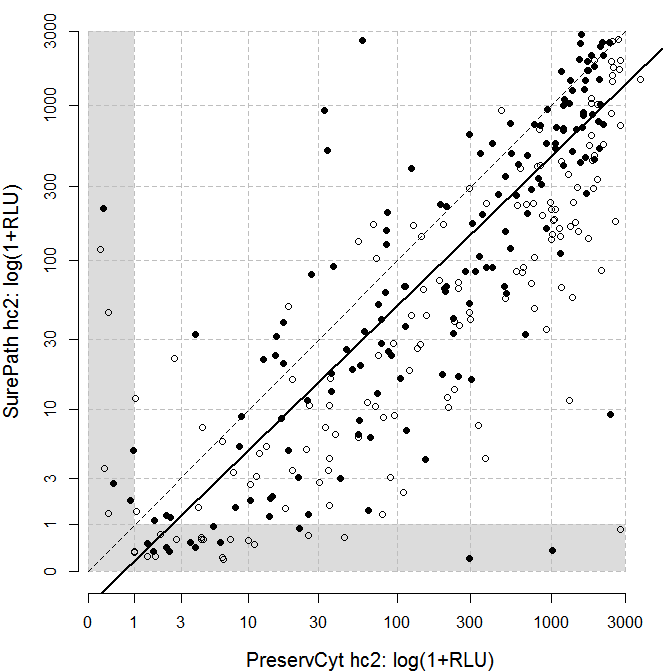


**Supplementary Figure S3. Scatterplot of Realti*m*e Ct values for samples that are amplified for at least one test. The solid line is the regression line for SurePath regressed on PreservCyt adjusted for sample order. The dashed line is the 45 degree line. Samples with PreservCyt first are open and those with SurePath first are solid. Shaded area indicates values below the positivity cut-off.**


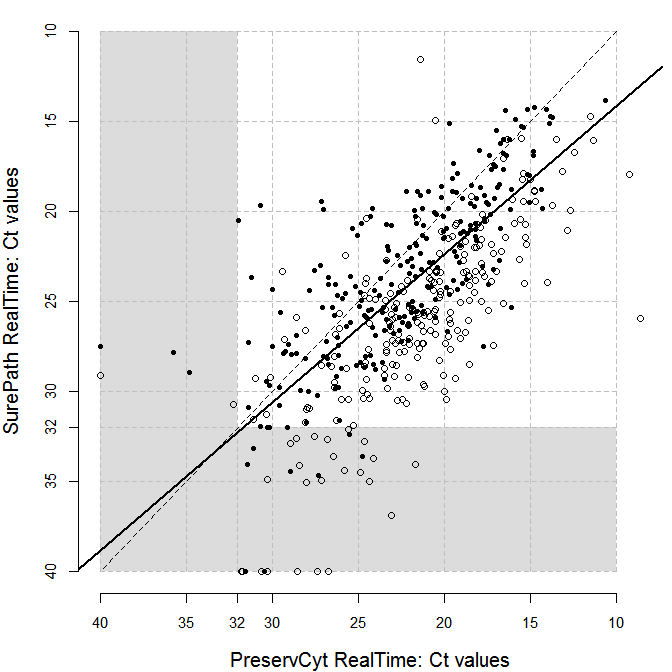


**Supplementary Figure S4. Scatterplot of Onclarity Ct values for samples that are amplified for at least one test. The solid line is the regression line for SurePath regressed on PreservCyt adjusted for sample order. The dashed line is the 45 degree line. Samples with PreservCyt first are open and those with SurePath first are solid. Shaded area indicates values below the positivity cut-off.**


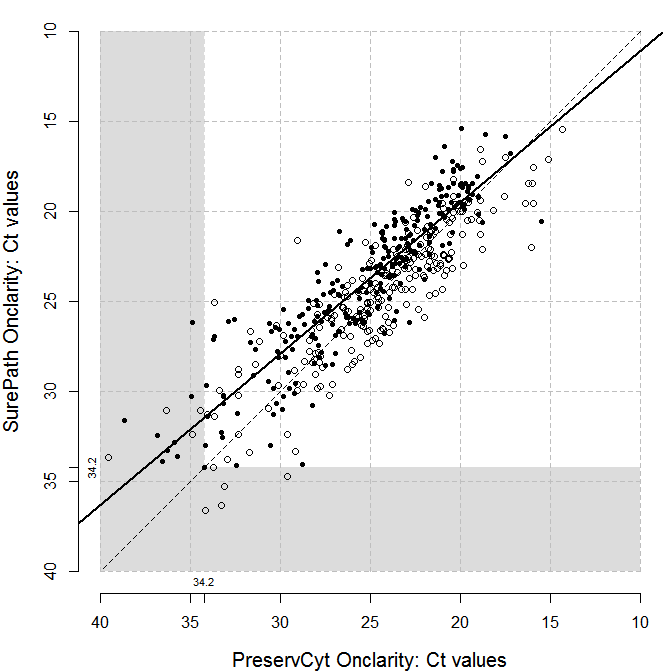


**Supplementary Figure S5. Scatterplot of PapType RIU values. The solid line is the regression line for SurePath regressed on PreservCyt adjusted for sample order. The dashed line is the 45 degree line. Samples with PreservCyt first are open and those with SurePath first are solid. Shaded area indicates values below the positivity cut-off.**


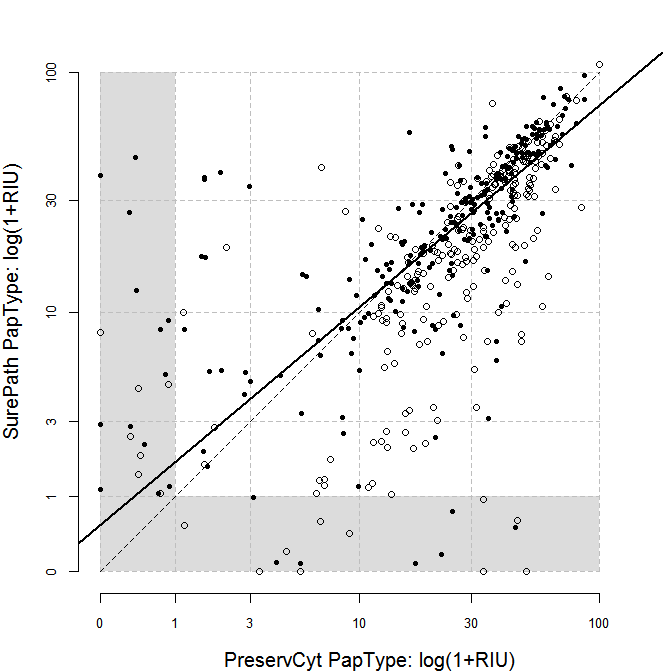


**Supplementary Figure S6. Scatterplot of Aptima RIU values. The solid line is the regression line for SurePath regressed on PreservCyt adjusted for sample order. The dashed line is the 45 degree line. Samples with PreservCyt first are open and those with SurePath first are solid. Shaded area indicates values below the positivity cut-off.**


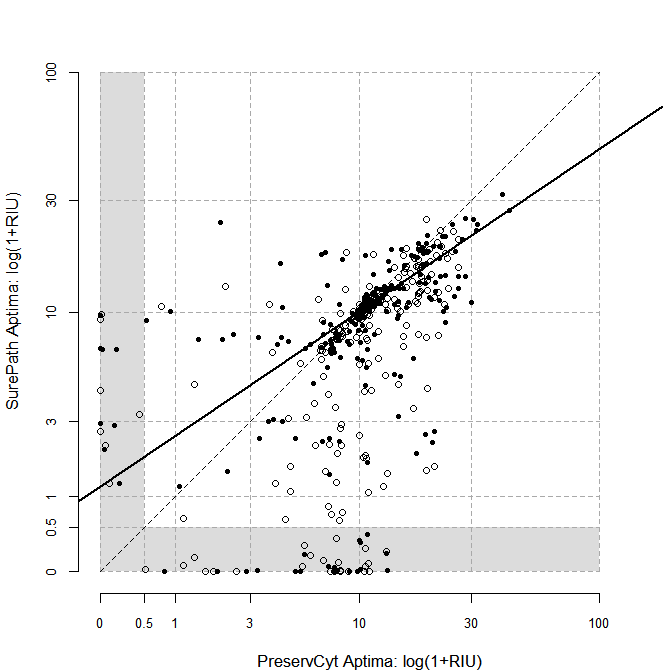


**Supplementary Figure S7. Scatterplot of OncoHealth ROD values. The solid line is the regression line for SurePath regressed on PreservCyt adjusted for sample order. The dashed line is the 45 degree line. Samples with PreservCyt first are open and those with SurePath first are solid. Shaded area indicates values below the positivity cut-off.**


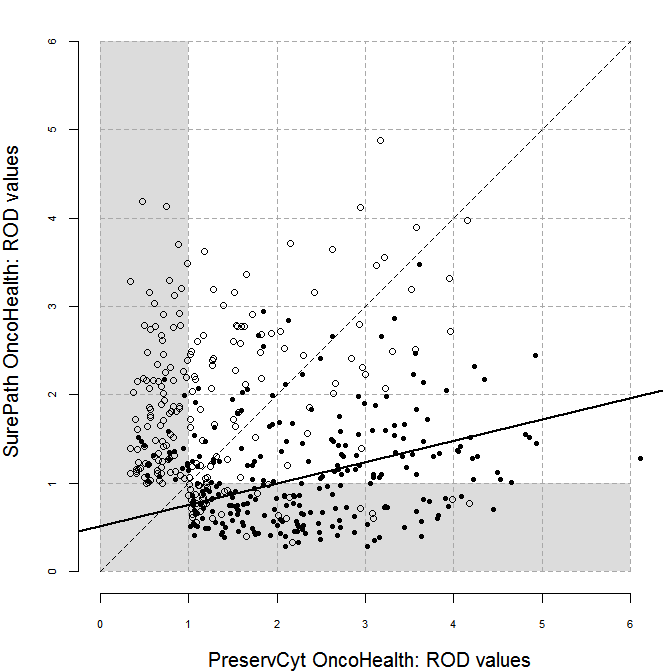

Supplement: Supplementary file 1 [file mmc1.doc]
